# Detection of Carbapenem Resistance of *Proteus mirabilis* Strains Isolated from Foxes, Raccoons and Minks in China

**DOI:** 10.3390/biology11020292

**Published:** 2022-02-11

**Authors:** Penghao Lv, Guijuan Hao, Yanli Cao, Lulu Cui, Guisheng Wang, Shuhong Sun

**Affiliations:** 1 Department of Preventive Veterinary Medicine, College of Veterinary Medicine, Shandong Agricultural University, Taian 271018, China; 2019110426@sdau.edu.cn (P.L.); sdaucyl@163.com (Y.C.); 2021010090@sdau.edu.cn (L.C.); 2 Shandong Animal Disease Prevention and Control Center, Taian 261500, China

**Keywords:** *Proteus mirabilis*, antimicrobial resistance, carbapenem resistance, virulence genes

## Abstract

**Simple Summary:**

*Proteus* is found abundantly in soil and water, and it has been known to cause human urinary tract infections and food poisoning. Currently, the opportunistic pathogen *Proteus mirabilis* (*P. mirabilis*) is also found to be an emerging threat to animals, such as birds, fish, dogs, etc. In this study, we examined the antibiotic resistance genes and virulence genes of *P. mirabilis* isolates from raccoons, foxes and minks. Among a total of 53 *P. mirabilis* isolates, the proportion of bacteria resistant to three or more antibiotic classes was up to 73.58%, and the detection rate of carbapenem-resistant *P. mirabilis* isolates was up to 71.7%, putting human health at risk. The close evolutionary relationship between *P. mirabilis* isolates from animals and the farm environment suggested that multidrug-resistant *P. mirabilis* from animals could pose a great environmental threat. In addition, the carriage rate of virulence-associated genes was not positively correlated with *P. mirabilis* pathogenicity in a *Galleria mellonella* model, highlighting the importance of further understanding the virulence of *P. mirabilis* in future studies.

**Abstract:**

*Proteus mirabilis*, an opportunistic pathogen, is found to be an emerging threat to both animals and humans for a variety of infections. However, the characteristics of *P. mirabilis* infections from foxes, raccoons and minks remain unclear. In this context, we identified the antibiotic resistance genes and virulence genes of *P. mirabilis* isolates from foxes, raccoons and minks in China. Most isolates showed resistance to florfenicol (90.57%), trimethoprim-sulfamethoxazole (73.58%), and imipenem (71.70%). A total of 73.58% of isolates were resistant to antibiotics from at least three or more classes, and were categorized as multi-drug resistant. A total of 33.33% of the isolates were resistant to antibiotics from seven classes. The most prevalent resistant were *sul1* (94.34%), followed by *floR*, *bla*_TEM_, *aac(6′)Ib-cr* and *bla*_OXA-1_ with the detection rate of 88.68%, 83.02%, 71.70% and 60.38%, respectively. Among the 51 *P. mirabilis* isolates that were resistant to beta-lactam antibiotics, all isolates carried at least one beta-lactam gene. In addition, *bla*_NDM_ and *bla*_OXA-24_ genes were firstly reported in carbapenem-resistant *P. mirabilis* isolates from foxes, raccoons and minks. All isolates exhibited the virulence genes *ureC*, *zapA*, *pmfA*, *atfA* and *mrpA*. *P. mirabilis* isolates carrying all detected 10 virulence genes from different animal species showed different lethal abilities in a *G. mellonella* larvae model. More importantly, the profiles of antibiotic resistance genes of isolates from fur animals and the environment were generally similar, and phylogenetic analysis showed that the *P. mirabilis* isolates from farm environment samples may have close relatedness with that from animals.

## 1. Introduction

*Proteus mirabilis* (*P. mirabilis*), a Gram-negative bacterium with flagella and fimbriae, is widely found in water, soil, and the intestinal microbiota of animals and humans. As an opportunistic pathogen, *P. mirabilis* is usually harmless to human health [1]. However, when the body’s immunity is weakened, *P. mirabilis* may move to non-intestinal sites and cause serious diseases, such as cystitis, sepsis, peritonitis and meningitis [2,3]. After *Escherichia coli* (*E. coli*) and *Klebsiella pneumoniae* (*K*. *pneumoniae*), *P. mirabilis* is the third most common cause of human urinary tract infections [4]. *P. mirabilis* is also involved in nosocomial outbreaks in neonates in India [3]. In addition, although *Proteus* does not belong to the foodborne pathogens, some food poisoning cases associated with *P. mirabilis* have been reported [5,6]. Gong et al. [6] demonstrated that *P. mirabilis* from humans could induce the symptoms of both vomiting and diarrhea in a mouse model and show high gastrointestinal pathogenicity. Comparative genomic analysis showed that *P. mirabilis* may acquire horizontally from other microbial genomes toxin genes to exert digestion tract infection and toxicity [7]. Currently, the opportunistic pathogen *P. mirabilis* is found to be an emerging threat to animals. It was reported that *P. mirabilis* could cause large-scale mortality of fish without association with any other pathogens [8]. In addition, *P. mirabilis* infections have been reported in birds with reproductive failure [9], cattle and fowl with diarrhea [10,11] and dogs with chronic otitis externa [12]. 

Carbapenems, such as imipenem and meropenem, are bactericidal beta-lactam antimicrobials with proven efficacy in severe infections caused by extended spectrum beta-lactamases (ESBLs, e.g., TEM, SHV, CTX-M enzymes) producing bacteria [13]. Carbapenem resistance occurs mainly among Gram-negative pathogens such as *Enterobacteriaceae*, *Pseudomonas* spp. and *Acinetobacter* spp., which is mainly due to the production of carbapenemase including some class A beta-lactamases (e.g., KPC, GES), class B metallo-beta-lactamases (e.g., NDM, IMP) and class D carbapenemases (e.g., OXA-23, -24, -48, -51, and -58) [14]. KPC-type producing *P. mirabilis* was identified for the first time in the blood culture of a diabetic patient in the United States in 2008; since then, KPC-producing *P. mirabilis* isolates have been found in humans in many countries [15,16,17]. NDM-producing *P. mirabilis* isolates have been isolated from commercial broilers in slaughterhouses, ducks, wildlife, and hospitalized patients in China [18,19,20,21]. Among the carbapenem-hydrolyzing class D beta-lactamases, OXA-48, the most frequently identified in *Enterobacterales*, has only been very rarely reported in *Proteus* spp., whereas OXA-23 and OXA-58, exclusively identified in *Acinetobacter* species, have recently been increasingly identified in *P. mirabilis* from hospital settings, the community, and cattle feces [17,22,23]. In addition, the *bla*_OXA-24_ has also been identified in *P. mirabilis* isolates from hospital clinical specimens in Algeria [22]. Since *P. mirabilis* is intrinsically resistant to tetracyclines and polymyxins, the emergence of multidrug-resistant, or even extensively drug-resistant *P. mirabilis* isolates carrying carbapenemase genes complicated the clinical treatment of bacterial infections, causing a significant public health concern. Most previous studies were focused on the prevalence of antimicrobial resistance of *P. mirabilis* in human urinary tract infections, and chicken, beef, and pork meat [19,21,23,24]. However, there are few reports on the characteristics of *P. mirabilis* from foxes, raccoons and minks. 

The present study aimed to characterize the antibiotic resistance profiles of 53 *P. mirabilis* isolates obtained from fox, raccoon and mink farms in China, and to compare the genotypic and phenotypic characteristics of antimicrobial resistance and virulence factors in these isolates.

## 2. Materials and Methods

### 2.1. Bacterial Isolates and Growth Conditions

A total of 53 *P. mirabilis* isolates were used in this study. They were isolated from fox, raccoon, or mink farms in different cities of Shandong Province, China between October 2019 and November 2020 (Table 1). Detailed information on these *P. mirabilis* isolates is provided in Tables S1 and S2 in the Supplementary Materials. The standard *Salmonella enterica* serovar Enteritidis (*S.* Enteritidis) strain CVCC3377 was purchased from the China Veterinary Culture Collection Center (Beijing, China). All isolates were streaked on xylose lysine deoxycholate (XLD) agar plates at 37 °C for 16~24 h, and then colonies were inoculated in Luria–Bertani (LB) medium. Cultures were grown at 37 °C and 180 rpm to the stationary phase for subsequent experiments.

### 2.2. Antimicrobial Susceptibility Testing

The CLSI disk diffusion method was used in this study to examine antibiotic resistance of *P. mirabilis* isolates to 11 antibiotics from 7 classes, including cefepime (FEP, 30 μg), cefotaxime (CTX, 30 μg), ceftazidime (CAZ, 30 μg), gentamicin (GM, 10 μg), imipenem (IPM, 10 μg), ampicillin (AMP, 10 μg), streptomycin (STR, 10 μg), ofloxacin (OFX, 5 μg), enrofloxacin (ENR, 15 μg), and trimethoprim-sulfamethoxazole (SXT, 25 μg). In brief, 0.5 McFarland *P. mirabilis* inoculum from liquid culture was spread onto Mueller–Hinton agar plates (Hopebiol), and the antibiotic discs were dispensed on the agar. Plates were incubated at 35 °C air incubator for 16 to 18 h. *E. coli* (ATCC 25922) was used as a quality control strain. The results were interpreted based on the Clinical and Laboratory Standards Institute (CLSI) M100-S27 guideline and CLSI Supplement VET01S [25,26]. The broth microdilution method was used for the determination of the minimum inhibitory concentrations (MICs) of *P. mirabilis* isolates to florfenicol (FFC). *P. mirabilis* isolates resistant to one or more antibiotics in three or more antibiotic classes were defined as multidrug-resistant (MDR).

### 2.3. Detection of Antimicrobial Resistance Genes

A total of 31 antimicrobial resistance genes in 5 categories, beta-lactam resistance genes (*bla*_SHV_, *bla*_OXA-1_, *bla*_OXA-23_, *bla*_OXA-24_, *bla*_OXA-58_, *bla*_OXA-48_, *bla*_CTX-M_, *bla*_TEM_, *bla*_PSE_, *bla*_KPC_, *bla*_NDM_, *bla*_IMP_, *bla*_VIM_) [27,28,29,30,31], aminoglycoside resistance genes (*aaC1*, *aaC2*, *aaC3, aadA*, *aadB*, *aphA6*, *aac (6′)-Ib-cr*) [32,33,34,35], quinolone resistance genes (*qnrA*, *qnrB*, *qnrC*, *qnrS*, *oqxA*, *aac (6′)-Ib-cr*) [27,35], folate pathway antagonist resistance genes (*sul1*, *sul2*, *sul3*) and phenicol resistance genes (*floR*, *cmlA*) were identified by PCR with the primers in Table A1 as previously described [27,36,37,38]. Bacterial DNA samples were prepared by the boiling method with a modification [39]. Briefly, approximately 10^8^ CFU/mL cells in ddH_2_O were boiled at 100 °C for 10 min in a water bath, then cells were centrifuged for five minutes at 1000 rpm and the supernatants were used for the PCR directly. The PCR was carried out in a 25 µL reaction mixture containing 12.5 µL of 2× Taq Master Mix (Vazyme, Nanjing, China), 1 µL of each primer (10 pmol), 9.5 µL of ddH_2_O and 1 µL of DNA template using a T100TM Thermal Cycler (BIO-RAD, Hercules, CA, USA). ddH_2_O was used as a negative control. PCR products were analyzed by 1% agarose gel electrophoresis at 100 V for 20 min and imaged.

### 2.4. Detection of Virulence Genes

The virulence genes *mrpA*, *pmfA*, *atfA*, *atfC* (fimbriae), *ureC* (urease), *zapA* (protease), *ucaA* (adhesin), *rsbA* (migration), *rsmA* (repressor of secondary metabolites) and *hmpA* (hemolysin) of *P. mirabilis* were identified with the previous primers and described PCR reaction system (Appendix A Table A2) [11,40], the bacterial DNA and PCR reaction mixture were prepared as described above.

### 2.5. Infection of Galleria Mellonella Larvae

In this study, the larvae of the *Galleria mellonella* (*G. mellonella*) wax moth was used to assess the pathogenicity of *P. mirabilis* as described previously with modifications [41]. Stationary bacterial cells were washed with PBS and then diluted in PBS to an optical density at OD_600_ of 1, which corresponds to approximately 1 × 10^9^ CFU/mL. For 50% lethal dose (LD_50_) experiments, a series of 10-fold serial dilutions containing 10^4^ to 10^8^ CFU in PBS were injected into *G. mellonella* larvae. After surface disinfection using 70% ethanol, a Microliter Syringe (Shanghai Gaoge, Shanghai, China) was used to inject 10 μL aliquots of the inoculum into the hemocoel of each larvae via the last left proleg [42]. A group of 10 larvae were injected with 10 μL of PBS in parallel to ensure that death was not due to injection trauma. Larvae were placed in 9.2 cm Petri dishes and kept at 37 °C in the dark. Insects were considered dead when they displayed no movement in response to touch. The number of dead larvae was scored at 6, 12, and 24 h post-infection. For each strain, at least three independent experiments were performed, and LD_50_s were calculated according to the formula of Käber [43].

### 2.6. Determination of In Vivo Bacterial Loads

Each group containing 15 larvae was infected with approximately 1 × 10^3^ CFU per larva of *P. mirabilis* isolates (F13, R8 and M1) or *S.* Enteritidis CVCC3377, respectively. Three insects in each group were collected at different post-infection time points (6 h and death time within 12 h) to determine bacterial loads. After surface disinfection with 70% ethanol, larvae were homogenized in 2 mL of PBS by use of a High-Speed KZ-II Basic Homogenizer (Servicebio, Wuhan, China). Serial dilutions of the homogenates in PBS were plated on XLD agar, and colonies were counted after incubation at 37 °C for 24 h. Three independent experiments were performed. 

### 2.7. Phylogenetic Tree Analysis

The 16S ribosomal RNA (16S rRNA) gene sequences of 53 *P. mirabilis* isolates were amplified with primers 27F and 1492R [44]. The PCR products were sequenced and identified using BLAST algorithms against the NCBI databases. A total of 8 representative *P. mirabilis* isolates from different isolation sources (chicken, snake, milk, etc.) were selected to construct a phylogenetic tree. Multiple alignments of the 16S rRNA gene sequences were conducted with the ClustalW algorithm, and phylogenetic trees were constructed by MEGA 7.0 software (Pennsylvania State University, College Town, PA, USA) using the Neighbor-Joining method [45].

### 2.8. Data Availability

The partial 16S rRNA gene sequences of 53 *P. mirabilis* isolates were all deposited in the NCBI database under the GenBank accession numbers OL629182–OL629203 (F1–F22), OL629209–OL629226 (R1–R18) and OL629229–OL629241 (M1–M13). 

## 3. Results

### 3.1. Antimicrobial Susceptibility of P. mirabilis Isolates

**Figure 1:**
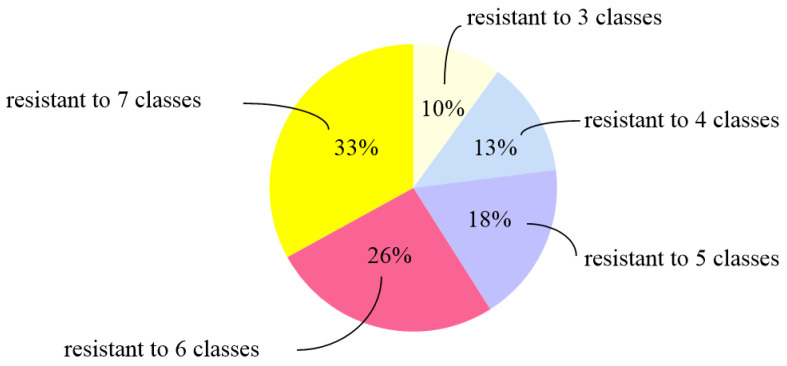
The proportion of MDR *P. mirabilis* isolates to different classes of antibiotics used in this study.

As shown in Table 2, all isolates were sensitive to cefepime (53/53, 100%), and most isolates were sensitive to ceftazidime (48/53, 90.57%) and ofloxacin (44/53, 83.02%). Most isolates were resistant to florfenicol (48/53, 90.57%), trimethoprim-sulfamethoxazole (39/53, 73.58%), imipenem (38/53, 71.70%), enrofloxacin (34/53, 64.15%), ampicillin (33/53, 63.46%), streptomycin (32/53, 60.38%), gentamicin (27/53, 50.94%), and cefotaxime (26/53, 49.06%). We found that the proportion of MDR isolates was 73.58% (39/53). Among the 39 MDR *P. mirabilis* isolates, isolates resistant to seven classes of antibiotics accounted for the highest proportion (33.33%, 13/39) (Figure 1), of which nine were from raccoons and four were from foxes.

**Figure 2:**
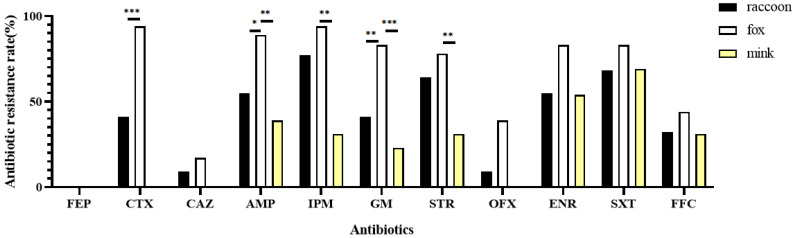
Comparison of the antibiotic resistance rates of *P. mirabilis* isolates between raccoons, foxes and minks. The difference was analyzed by chi-squared test. * *p* < 0.05; ** *p* < 0.01; *** *p* < 0.001.

The isolates from the fox farm had generally higher resistance rate than the isolates from raccoon and mink farms (Figure 2). Among the *P. mirabilis* isolates from raccoons and foxes, the significantly different resistance rates to antibiotics were as follows, respectively: to CTX, 40.9% and 94.4% (*p* < 0.001); to AMP, 54.5% and 88.9% (*p* < 0.05); to GM, 40.9% and 83.3% (*p* < 0.01). Meanwhile, by comparing the antibiotic resistance rates between the isolates from minks and foxes, the difference was mainly in AMP (*p* < 0.01), IMP (*p* < 0.01), GM (*p* < 0.001) and STR (*p* < 0.01). The resistant rates of *P. mirabilis* isolates from raccoons and foxes to CTX, CAZ and OFX were 9.1%∼94%, but no isolate from mink showed resistance to the above three antibiotics. No difference was found in ENR, SXT and FFC resistance among the isolates from the above three kinds of animals.

### 3.2. Antimicrobial Resistance Genes of P. mirabilis

**Figure 3:**
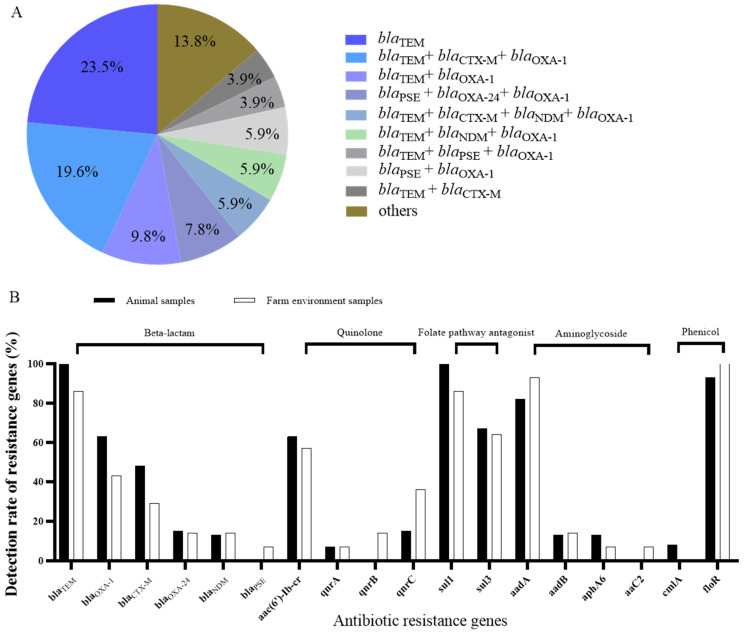
(**A**) Beta-lactam antibiotics resistance genes among the 53 *P. mirabilis* isolates. (**B**) Comparison of the detection rate of resistance genes in *P. mirabilis* isolates from animal samples and farm environment samples.

The most frequently detected antimicrobial resistance gene was *sul1* (94.34%, 50/53), followed by *floR*, *bla*_TEM_, *aac(6′)Ib-cr* and *bla*_OXA-1_, with the detection rate of 88.68% (47/53), 83.02% (44/53), 71.70% (38/53) and 60.38% (32/53), respectively (Table 3). Among the 14 beta-lactamase genes detected, *bla*_TEM_ was the most prevalent beta-lactamase gene (83.02%), followed by *bla*_OXA-1_ (60.38%), *bla*_CTX-M_ (32.08%), *bla*_PSE_ (24.53%), *bla*_OXA-24_ (15.09%), and *bla*_NDM_ (13.21%). Among the 51 *P. mirabilis* isolates that were resistant to beta-lactam antibiotics, all isolates carried at least one related drug resistance gene, of which most isolates (23.5%) carried only the *bla*_TEM_ gene, followed by isolates harboring *bla*_TEM_, *bla*_CTX-M_ and *bla*_OXA-1_ (19.6%) and isolates harboring both *bla*_TEM_ and *bla*_OXA-1_ (9.8%) (Figure 3A). For quinolone resistance genes, the detection rate of *aac(6′)-Ib-cr*, *qnrA*, *qnrB* and *qnrC* was 71.70%, 5.66%, 22.64% and 15.09%, respectively, but *qnrS* and *oqxA* were not detected. Most isolates carry the folate pathway antagonist resistance gene *sul1* (94.34%), followed by *sul3* (54.72%) and *sul2* (11.32%), respectively. Among phenicol resistance genes, the detection rate of *floR* was up to 88.68%, followed by *cmlA* (5.66%). In addition, four aminoglycoside resistance genes (*aaC2*, *aadA*, *aadB* and *aphA6*) were detected, accounting for 9.43%, 83.02%, 13.23% and 11.32%, respectively. 

By comparing the antimicrobial resistance genes from fox, raccoon and mink farms, we found that gene *bla*_TEM_, *bla*_OXA-1_, *aac(6′)-Ib-cr*, *sul1*, and *floR* were common. However, *bla*_CTX-M_, *qnrA* and *qnrC* were only detected in *P. mirabilis* isolates from raccoons and foxes. In addition, the genes *bla*_PSE_, *qnrB*, *aaC2*, *sul2* and *cmlA* were specific for the isolates from minks. These results show the diversity of antibiotic resistance genes among different farms. 

We also compared the detection rate of antibiotic resistance genes between samples from animals (feces, throat, anal and carcass) and the farm environment (feed and soil), and found that the detection rates of antibiotic resistance genes were generally similar (Figure 3B). The proportions of *bla*_TEM_-positive, *sul1*-positive and *floR*-positive isolates from animal samples and farm environment samples were all above 85%. Although *bla*_TEM_*, bla*_OXA-1_, *bla*_CTX-M_, *bla*_OXA-24_, *bla*_NDM_, *aac(6′)-Ib-cr*, *qnrA*, *qnrC*, *sul1*, *sul3*, *aadA*, *aadB*, *aphA6* and *floR* genes were prevalent in both animal samples and farm environment samples, no significant difference (*p* > 0.05) was found between the positive rates of the two groups of samples. 

### 3.3. Virulence Genes of P. mirabilis Isolates

Among the 10 virulence genes, *ureC*, *zapA*, *pmfA*, *atfA* and *mrpA* genes were all detected among 53 *P. mirabilis* isolates, followed by both *atfC* and *hmpA* genes, both with a detection rate of 98.11%, *rsmA* at 94.34%, *rsbA* at 71.70% and *ucaA* at 45.28% (Table 4). 

### 3.4. Pathogenicity of P. mirabilis Isolates

#### 3.4.1. Pathogenicity Comparison of *P. mirabilis* Isolates from Different Animal Sources

To determine the pathogenicity of *P. mirabilis* isolates from different animal sources, three *P. mirabilis* isolates (F13, R8 and M1) that carry all detected 10 virulence genes were selected. The LD_50_ of *P. mirabilis* F13, R8, and M1 in *G. mellonella* larvae model were 2.5 × 10^4^ CFU/larvae, 2.0 × 10^4^ CFU/larvae and 3.9 × 10^5^ CFU/larvae at 6 h post infection, respectively (Table 5). The results indicate that the virulence of *P. mirabilis* M1 from minks seemed to be weaker than F13 from foxes and R8 from raccoons.

**Figure 4:**
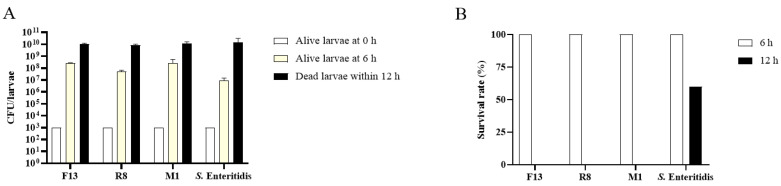
Comparison of pathogenicity between three *P. mirabilis isolates* from different animal sources and *S.* Enteritidis. (**A**) The bacterial loads in larvae infected with *P. mirabilis* and *S.* Enteritidis. (**B**) Survival rate in larvae infected with *P. mirabilis* and *S.* Enteritidis at 6 and 12 h post infection. Each group was infected with approximately 1 × 10^3^ CFU per larva of *P. mirabilis* isolates (F13, R8 and M1) or *S.* Enteritidis CVCC3377, respectively. Three infected larvae from each group were pooled and homogenized, and the numbers of CFU were determined by plating. The survival rates in each group were calculated at 6 and 12 h post-infection. *n* = 15.

To further determine whether *G. mellonella* mortality is associated with the growth of bacteria in infected larvae, larvae were injected with 10^3^ CFU of *P. mirabilis*. As shown in Figure 4A, infection of *G. mellonella* with the F13, R8, and M1 strain resulted in a 10^4^~10^5^-fold increase in CFU at 6 h post infection, which was followed by further increases in the bacterial numbers of dead larvae within 12 h post infection. These results are similar to that of larvae infected with pathogenic *S.* Enteritidis CVCC3377, which caused a remarkable increase in bacterial numbers in *G. mellonella* over time. At 12 h post infection, larvae in *P. mirabilis* infection group were all dead, whereas 60% larvae survived in the *S.* Enteritidis infection group (Figure 4B). The results demonstrate that *P. mirabilis* isolates from fox, mink and raccoon farms could replicate rapidly in vivo and displayed high virulence in *G. mellonella* larvae model. 

#### 3.4.2. Pathogenicity of *P. mirabilis* Isolates with Different Virulence Genes

To evaluate the effect of virulence genes on the pathogenicity of *P. mirabilis* isolates, three isolates, which were all isolated from fox feces but with different virulence genes, were selected to compare their virulence in a *G. mellonella* model. As shown in Table 6, the LD_50_ of F2, F3, and F6 isolates at 6 h post infection was 2.0 × 10^4^ CFU/larvae, 7.9 × 10^3^ CFU/larvae and 4.0 × 10^3^ CFU/larvae, respectively. Although strain F2 carried all detected virulence genes, its LD_50_ was 2.53- and 5-fold higher than that of strain F3 lacking *ucaA*, *rsbA* and *rsmA*, and strain F6 lacking *ucaA*. The results indicate that the carriage rate of virulence-associated genes was not positively correlated with *P. mirabilis* pathogenicity in *G. mellonella* larvae.

### 3.5. Phylogenetic Analysis of P. mirabilis Isolates

**Figure 5:**
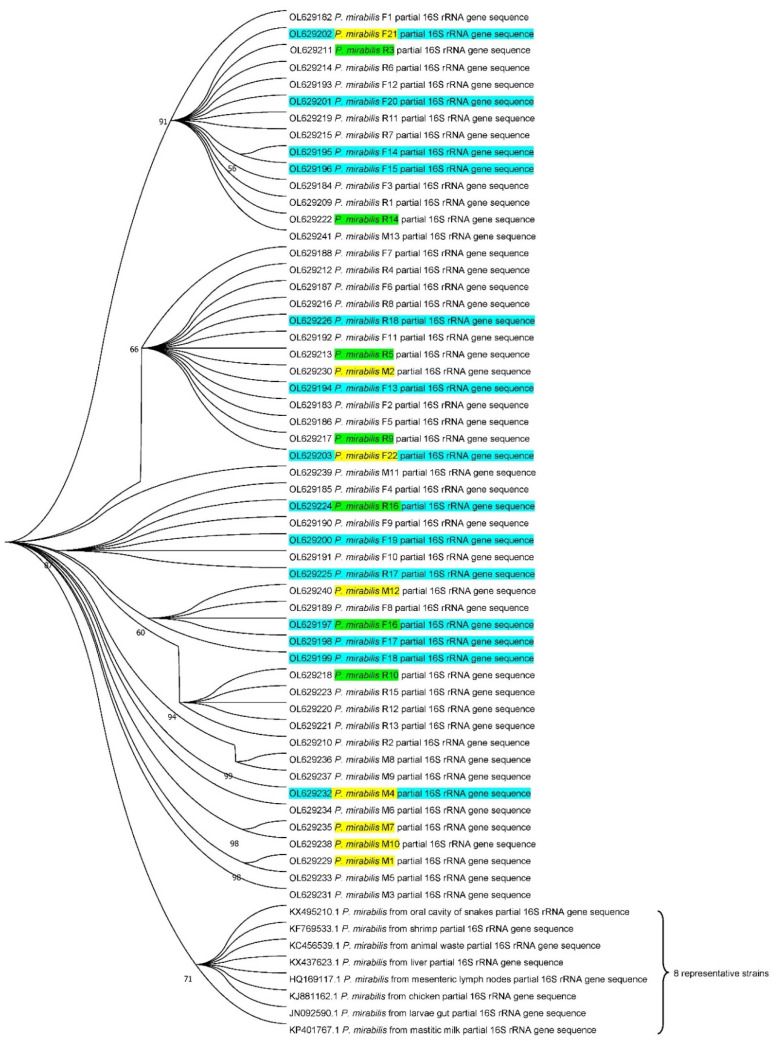
Phylogenetic tree constructed based on 16S rRNA sequences of 61 *P. mirabilis* isolates using the neighbor-joining method. Eight representative isolates from different sources are labeled with big parentheses, 14 isolates from farms environment in the present study are marked in turquoise, and isolates harboring *bla*_NDM_ or *bla*_OXA-24_ are marked in green or yellow, respectively. All sequences were aligned using ClustalW, then the aligned data were evaluated by the neighbor-joining approach using MEGA7 software with 2000 replications of bootstrap. Branches corresponding to partitions reproduced in less than 50% bootstrap replicates are collapsed. There were a total of 1337 positions in the final dataset.

The 53 *P. mirabilis* isolates and the selected eight representative isolates of *P. mirabilis* 16S rRNA gene sequences obtained on NCBI were used to perform phylogenetic analysis to understand the evolutionary relationship among the *P. mirabilis* isolates (Figure 5). The results show that the sequence similarity between the 53 isolates and eight representative bacteria was 99.5~99.9%, but the *P. mirabilis* isolates from fur farms have far lower relatedness with the representative *P. mirabilis* isolates from shrimp, chick, mastitic milk, etc. The 22 *P. mirabilis* isolates from foxes and 18 isolates from raccoons were co-clustered into three branches, but the 13 *P. mirabilis* isolates from minks formed many clades in the evolutionary phylogeny, indicating there may be multiple origins of *P. mirabilis* isolates from minks. Moreover, the isolates from animal samples and farm environment samples were distributed across different branches. This pattern suggested that the isolates from farm environment samples had close relatedness with those from animal samples.

## 4. Discussion

Bacterial antibiotic resistance is a global public health threat, and one important cause of it is the multidrug bacterial isolates of animal origins acting as an important source for human infections [46,47,48]. We investigated the characteristics of 53 *P. mirabilis* isolates from fox, raccoon and mink farms—the results show that the proportion of MDR isolates among all the *P. mirabilis* isolates was up to 73.58%, similar to that among the 32 *P. mirabilis* isolates from chicken carcasses in a poultry slaughterhouse in Brazil (78.13%) [47]. ESBL production is significantly associated with mortality in patients with bacteremia caused by *P. mirabilis* [49]. In this study, 51 of the 53 *P. mirabilis* isolates were resistant to beta-lactam antibiotics and most isolates exhibited two or more beta-lactamase genes. The most common ESBL-encoding gene among the 53 *P. mirabilis* isolates from foxes, raccoons and minks is *bla*_TEM_ (83.02%), which is spread worldwide and is now found in many different species of the order of *Enterobacterales* [17]. Carbapenem resistance rates are typically extremely low in clinical *P. mirabilis* isolates; however, approximately 71.70% of isolates from foxes, raccoons and minks were resistant to imipenem. Among the nine detected carbapenemase genes, the class D carbapenemase gene *bla*_OXA-24_ (15.09%) and the class B metallo-beta-lactamase *bla*_NDM_ (13.21%) were found for the first time in *P. mirabilis* isolates from foxes, raccoons and minks. Kang et al. found that 16.67% (6/54) of the *P. mirabilis* isolates from wild animals were resistant to meropenem, and they mainly carried the carbapenem-resistance genes *bla*_OXA-1_ and *bla*_NDM-1_ [19]. For the 35 *P. mirabilis* isolates obtained from 240 duck samples in Egypt, only three strains were carbapenem-resistant, two strains harbored the *bla*_NDM-1_ gene, and one strain carried the *bla*_KPC_ gene [21]. The presence of *bla*_NDM-1_ in *Proteus* spp. clinical isolates is still episodic in China and many other countries [17]. However, the *bla*_NDM-1_ gene had been identified on a genomic island in *P. mirabilis* recovered from the urine of a hospitalized patient in France in 2012 [50]. The *bla*_OXA-24_ gene, mainly detected in *Acinetobacter *baumannii,** were shown to appear in reservoirs including livestock, companion animals, and the environment [17,51]. However, the *bla*_OXA-24_ gene is only detected in clinical strains of *P. mirabilis* in the United States [22]. To the best of our knowledge, *P. mirabilis* isolates harboring *bla*_OXA-24_ were identified for the first time from animal origins. Some other mechanisms including through porin mutation with or without decreased expression of penicillin binding proteins may contribute to the high imipenem-resistance rates of *P. mirabilis* isolates in this study. Moreover, the prevalence of beta-lactam-resistant *P. mirabilis* isolates from fur animals in this study and the similar antibiotic resistance profiling between *P. mirabilis* isolates from fur animals and farm environment suggested that MDR *P. mirabilis* isolates may likely spread from fur animals to the environment and potentially humans, posing a public health threat [52]. 

We also found increasing prevalence and diversity of some other antibiotic resistance genes in *P. mirabilis* from foxes, raccoons and minks. It was reported that *aac(6′)-Ib-cr* conferring quinolones and aminoglycosides resistance was common in *Escherichia coli* [53]. Here we showed that the detection rate of *aac(6′)-Ib-cr* among 53 *P. mirabilis* isolates was up to 71.7%, similar to the 63.2% among 19 *P. mirabilis* isolates from a Chinese hospital by Hu et al. [54]. The detection rates of quinolone resistance genes *qnrA* (5.66%) and *qnrB* (22.64%) were higher than the 0% from chicken carcasses in Brazil by Sanches et al. [19]. For the aminoglycoside resistance of *P. mirabilis* isolates in this study, another frequently encountered resistance gene was *aadA,* which may result in high resistance rates to streptomycin in these *P. mirabilis* isolates. These results indicate that *aac(6′)-Ib-cr* and *aadA* gene might be common in *P. mirabilis* isolates in foxes, raccoons and minks of China. Florfenicol is mainly used in veterinary medicine, a key gene for florfenicol and chloramphenicol resistance, *floR*, coding for an efflux protein of 12 transmembrane segments, can spread among bacteria of the same and different species or genera through horizontal gene transfer [55]. In this study, we found that 90.57% of *P. mirabilis* isolates were resistant to florfenicol, of which 77.36% carried the *floR* gene. So, the emergence and dissemination of florfenicol resistance among *P. mirabilis* isolates will limit the use of this antimicrobial for treating bacterial infections.

All *P. mirabilis* isolates harbored *ureC*, *zapA*, *pmfA*, *atfA* and *mrpA* virulence genes, which were also prevalent in the *P. mirabilis* isolates from chicken carcasses [47] and pork meat [24]. Among the 10 virulence genes, the detection rate of ucaA, a major fimbrial subunit that can enhance the adhesion of *P. mirabilis*, was the lowest [56]. However, we showed that the virulence of a *ucaA*-positive strain was about 5-fold lower than that of a *ucaA*-negative strain *in* *G. mellonella* larvae. Moreover, the carriage rate of virulence-associated genes in these *P. mirabilis* isolates did not correlate with higher pathogenicity in a *G. mellonella* model. Three *P. mirabilis* isolates carrying all detected 10 virulence genes from different animal species showed different lethal abilities in *G. mellonella* larvae. These *P. mirabilis* isolates could multiply rapidly in the hemolymph of the larvae, similarly to *S.* Enteritidis, resulting in a fierce but ineffective inflammatory response triggered by high bacterial burdens and hemocyte depletion [57]. These results suggest that the carriage rate of virulence genes in *P. mirabilis* isolates can only reflect their epidemic, the pathogenicity of bacteria should be evaluated by appropriate animal models.

## 5. Conclusions

In the present study, we firstly reported the emergence of carbapenem-resistant *P. mirabilis* isolates harboring *bla*_OXA-24_ (15.09%) or *bla*_NDM_ (13.21%) from foxes, raccoons and minks. All *P. mirabilis* isolates harbored *ureC*, *zapA*, *pmfA*, *atfA* and *mrpA* genes, but the carriage rate of these virulence-associated genes in the isolates did not correlate with higher pathogenicity in a *G. mellonella* model. Moreover, phylogenetic analysis showed that the *P. mirabilis* isolates from farm environment samples may have close relatedness with that from animals.

## Figures and Tables

**Table 1:** Fifty-three isolates of *P. mirabilis* from fox, mink and raccoon farms used in this study.

Isolates	Year of Isolation	Source	
		Animal	Farm Environment ^1^
F1-F22	2019	12 isolates from fox feces	9 isolates from soil samples 1 isolate from feed sample
R1-R18	2019	15 isolates from raccoon feces	3 isolates from soil samples
M1-M13	2020	6 isolates from mink feces, 2 isolates from carcass samples, 3 isolates from throat samples, 1 isolate from annal sample	1 isolate from feed sample

^1^ The soil samples were collected from the soil within 1 m around the animal cage, and the feed samples were collected from leftover animal feed in the feed trough. F1–F22 are from one fox farm, R1–R18 are from one raccoon farm and M1–M13 are from two mink farms in China.

**Table 2:** The sensitivity of 53 *P. mirabilis* isolates isolated from fox, raccoon and mink farms to 11 antibiotics.

Antibiotic Classes	Antibiotics	Number of Isolates		
		Resistant	Intermediate	Susceptible
Cephems	Cefepime (FEP)	0	1 (1.89%)	52 (98.11%)
	Cefotaxime (CTX)	26 (49.06%)	8 (15.10%)	19 (35.85%)
	Ceftazidime (CAZ)	5 (9.43%)	0	48 (90.57%)
Penicillins	Ampicillin (AMP)	33 (63.46%)	11 (20.75%)	9 (16.98%)
Carbapenems	Imipenem (IPM)	38 (71.70%)	0	15 (28.30%)
Aminoglycosides	Gentamicin (GM)	27 (50.94%)	1 (1.89%)	25 (47.17%)
	Streptomycin (STR)	32 (60.38%)	13 (24.53%)	8 (15.10%)
Quinolones	Ofloxacin (OFX)	9 (16.98%)	8 (15.09%)	36 (67.92%)
	Enrofloxacin (ENR)	34 (64.15%)	14 (26.42%)	5 (9.43%)
Folate pathway antagonists	Trimethoprim-sulfamethoxazole (SXT)	39 (73.58%)	1 (1.89%)	13 (24.53%)
Phenicols	Florfenicol (FFC)	48 (90.57%)	4 (7.55%)	1 (1.89%)

**Table 3:** Detection rates of drug resistance genes of 53 *P. mirabilis* isolates used in this study.

Name	Gene	Detection Rate			
		Raccoon	Fox	Mink	>Total
Beta-lactams	*bla*_TEM_	33.96%	41.51%	7.55%	83.02%
	*bla*_SHV_	0	0	0	0
	*bla*_PSE_	0	0	24.53%	24.53%
	*bla*_OXA-1_	32.08%	9.43%	18.87%	60.38%
	*bla*_OXA-23_	0	0	0	0
	*bla*_OXA-24_	0	3.78%	11.32%	15.09%
	*bla*_OXA-58_	0	0	0	0
	*bla*_OXA-48_	0	0	0	0
	*bla*_CTX-M_	18.87%	13.21%	0	32.08%
	*bla*_KPC_	0	0	0	0
	*bla*_NDM_	11.32%	1.89%	0	13.21%
	*bla*_IMP_	0	0	0	0
	*bla*_VIM_	0	0	0	0
Aminoglycosides	*aaC1*	0	0	0	0
	*aaC2*	0	0	9.43%	9.43%
	*aaC3*	0	0	0	0
	*aadA*	28.30%	32.08%	22.64%	83.02%
	*aadB*	7.56%	1.89%	3.78%	13.23%
	*aphA6*	0	5.66%	5.66%	11.32%
Quinolones	*aac(6′)-Ib-cr*	32.08%	15.09%	24.53%	71.70%
	*qnrA*	3.78%	1.89%	0	5.66%
	*qnrB*	0	0	22.64%	22.64%
	*qnrC*	9.43%	5.66%	0	15.09%
	*qnrS*	0	0	0	0
	*oqxA*	0	0	0	0
Folate pathway antagonists	*sul1*	33.96%	41.51%	18.87%	94.34%
	*sul2*	0	0	11.32%	11.32%
	*sul3*	32.08%	20.75%	1.89%	54.72%
Phenicols	*cmlA*	0	0	5.66%	5.66%
	*floR*	33.96%	41.51%	13.21%	88.68%

**Table 4:** Detection rate of 10 virulence genes of *P. mirabilis* isolates from different farms.

Gene	Fox	Raccoon	Mink	Total
	(*n* = 22)	(*n* = 18)	(*n* = 13)	(*n* = 53)
*ureC*	100%	100%	100%	100%
*zapA*	100%	100%	100%	100%
*pmfA*	100%	100%	100%	100%
*mrpA*	100%	100%	100%	100%
*atfC*	100%	94.44%	100%	98.11%
*atfA*	100%	100%	100%	100%
*ucaA*	45.45%	33.33%	61.54%	45.28%
*rsbA*	68.18%	61.11%	92.31%	71.70%
*rsmA*	95.45%	100%	84.62%	94.34%
*hmpA*	95.45%	100%	100%	98.11%

**Table 5:** The LD_50_s of three *P. mirabilis* isolates from different animal species in *G. mellonella* larvae model.

Isolates	Bacteria Dose (CFU/Larvae)	Dead			LD_50_ (6 h) (CFU/Larvae)
		6 h	12 h	24 h	
F13	1 × 10^6^	9	10	10	2.5 × 10^4^
	1 × 10^5^	6	10	10	
	1 × 10^4^	6	10	10	
	1 × 10^3^	0	10	10	
	1 × 10^2^	0	10	10	
R8	1 × 10^6^	9	10	10	2.0 × 10^4^
	1 × 10^5^	10	10	10	
	1 × 10^4^	3	10	10	
	1 × 10^3^	0	10	10	
	1 × 10^2^	0	10	10	
M1	1 × 10^6^	9	10	10	3.9 × 10^5^
	1 × 10^5^	0	10	10	
	1 × 10^4^	0	10	10	
	1 × 10^3^	0	9	10	
	1 × 10^2^	0	6	10	

**Table 6:** Pathogenicity of *P. mirabilis* isolates with different virulence genes from foxes in *G. mellonella* larvae.

Tested Strain	Virulence Gene			Bacteria Dose (CFU/Larvae)	Dead			LD_50_ (6 h) (CFU/Larvae)
	*ucaA*	*rsbA*	*rsmA*		6 h	12 h	24 h	
F2	+	+	+	1 × 10^6^	10	10	10	2.0 × 10^4^
				1 × 10^5^	7	10	10	
				1 × 10^4^	5	10	10	
				1 × 10^3^	0	1	3	
				1 × 10^2^	0	0	2	
F3	-	-	-	1 × 10^6^	10	10	10	7.9 × 10^3^
				1 × 10^5^	9	10	10	
				1 × 10^4^	7	10	10	
				1 × 10^3^	0	10	10	
				1 × 10^2^	0	8	10	
F6	-	+	+	1 × 10^6^	10	10	10	4.0 × 10^3^
				1 × 10^5^	10	10	10	
				1 × 10^4^	9	10	10	
				1 × 10^3^	0	10	10	
				1 × 10^2^	0	10	10	

## Data Availability

The original contributions generated for this study are included in the article, further inquiries can be directed to the corresponding authors.

## Supplementary Materials

The following are available online at www.mdpi.com/article/10.3390/biology11020292/s1, Table S1. Characteristics of virulence genes of 53 P. mirabilis isolates from fur animal farms in this study. Table S2. Characteristics of antibiotic resistance genes of 53 P. mirabilis isolates from fur animal farms in this study.

## Appendix A

**Table A1:** Primer sequence and fragment size of drug resistance genes.

Name	Gene	Sequence (5’-3’)	Size (bp)	Reference
Beta-lactams	*bla*_TEM_	F: ATAAAATTCTTGAAGACGAAA	643	[27]
		R: GACAGTTACCAATGCTTAATC		
	*bla*_SHV_	F: TTATCTCCCTGTTAGCCACC	860	[27]
		R: GATTTGCTGATTTCGCTCGG		
	*bla*_PSE_	F: TAGGTGTTTCCGTTCTTG	150	[28]
		R: TCATTTCGCTCTTCCATT		
	*bla*_OXA-1_	F: TCAACTTTCAAGATCGCA	591	[27]
		R: GTGTGTTTAGAATGGTGA		
	*bla*_OXA-23_	F: GATCGGATTGGAGAACCAGA	501	[30]
		R: ATTTCTGACCGCATTTCCAT		
	*bla*_OXA-24_	F: TTCCCCTAACATGAATTTGT	1024	[30]
		R: GTACTAATCAAAGTTGTGAA		
		R: TGGATTGCACTTCATCTTGG		
	*bla*_OXA-58_	F: TGGCACGCATTTAGACCG	507	[30]
		R: AAACCCACATACCAACCC		
	*bla*_OXA-48_	F: GCGTGGTTAAGGATGAACAC	438	[29]
		R: CATCAAGTTCAACCCAACCG		
	*bla*_CTX-M_	F: CGCTTTGCGATGTGCAG	550	[27]
		R: ACCGCGATATCGTTGGT		
	*bla*_KPC_	F: GACGGAAAGCTTACAAAAACTGACA	259	[31]
		R: CTTGTCATCCTTGTTAGGCG		
	*bla*_NDM_	F: GGTTTGGCGATCTGGTTTTC	181	[31]
		R: ATCCAGTTGAGGATCTGGGC		
	*bla*_IMP_	F: GGAATAGAGTGGCTTAATTCTC	275	[31]
		R: CAAGCTTCTATATTTGCGTCACC		
	*bla*_VIM_	F: GATGAGTTGCTTTTGATTGATACAGC	153	[31]
		R: CGCCCGAAGGACATCAA		
Quinolones	*qnrA*	F: ATTTCTCACGCCAGGATTTG	519	[27]
		R: GATCGGCAAAGGTCAGGTCA		
	*qnrB*	F: GATCGTGAAAGCCAGAAAGG	513	[27]
		R: ACGATGCCTGGTAGTTGTCC		
	*qnrC*	F: GGTTGTACATTTATTGAATC	666	[27]
		R: TCCACTTTACGAGGTTCT		
	*qnrS*	F: ACGACATTCGTCAACTGCAA	417	[27]
		R: TAAATTGGCACCCTGTAGGC		
	*oqxA*	F: GATCAGTCAGTGGGATAGTTT	670	[35]
		R: TACTCGGCGTTAACTGATTA		
	*aac(6′)-Ib-cr*	F: TTGCGATGCTCTATGAGTGGCTA	482	[27]
		R: CTCGAATGCCTGGCGTGTTT		
Aminoglycosides	*aaC1*	F: ACCTACTCCCAACATCAGCC	528	[32]
		R: ATATAGATCTCACTACGCGC		
	*aaC2*	F: ACTGTGATGGGATACGCGTC	482	[32]
		R: CTCCGTCAGCGTTTCAGCTA		
	*aaC3*	F: CACAAGAACGTGGTCCGCTA	185	[32]
		R: AACAGGTAAGCATCCGCATC		
	*aadA*	F: GTGGATGGCGGCCTGAAGCC	535	[34]
		R: AATGCCCAGTCGGCAGCG		
	*aadB*	F: ATGGACACAACGCAGGTC	495	[33]
		R: TTAGGCCGCATATCGCGACC		
	*aphA6*	F: ATGGAATTGCCCAATATTATTC	399	[33]
		R: TCAATTCAATTCATCAAGTTTTA		
Folate pathway antagonists	*sul1*	F: CTTCGATGAGAGCCGGCGG C	238	[37]
		R: GCAAGGCGGAAACCCGCGCC		
	*sul2*	F: CGGCATCGTCAACATAAC C	722	[36]
		R: GTGTGCGGATGAAGTCAG		
	*sul3*	F: AGATGTGATTGATTTGGGAGC	443	[38]
		R: TAGTTGTTTCTGGATTAGAGCCT		
Phenicols	*cmlA*	F: TGTCATTTACGGCATACTCG	900	[37]
		R: ATCAGGCATCCCATTCCCAT		
	*floR*	F: CACGTTGAGCCTCTATATGG	890	[27]
		R: ATGCAGAAGTAGAACGCGAC		

**Table A2:** Primers and annealing temperatures of 10 virulence genes.

Gene	Sequence (5′→3′)	Fragment	Annealing Temperature
*ureC*	F: GTTATTCGTGATGGGATGGG	375 bp	52 °C
	R: ATAAAGGTGGTTACGCCAG		
*mrpA*	F: ATTTCAGGAAACAAAAGATG	410 bp	39 °C
	R: TTCTTACTGATAAGACATTG		
*zapA*	F: ACCGCAGGAAAACATATAGCCC	493 bp	52 °C
	R: GCGACTATCTTCCGCATAATCA		
*atfA*	F: CATAATTTCTAGACCTGCCCTAGCA	365 bp	49 °C
	R: CTGCTTGGATCCGTAATTTTTAACG		
*atfC*	F: AGAAAGGGATCCTACAAATTAA	472 bp	49 °C
	R: TATAGCATGCATTTAAATTGCC		
*ucaA*	F: GTAAAGTTGTTGCGCAAAC	365 bp	49 °C
	R: TTGAGCCACTGTGGATACA		
*pmfA*	F: GGATCATCTATAATGAAACTG	534 bp	52 °C
	R: CTGATAATCAACTTGGAAGTT		
*rsbA*	F: TCGATTTCAGTGTTTGGCCAT	1647 bp	55 °C
	R: TCGATTTCAGTGTTTGGCCAT		
*rsmA*	F: TAGCGAGTGTTGACGAGTGG	562 bp	56 °C
	R: AGCGAGGTGAAGAACGAGAA		
*hmpA*	F: CCAGTGAATTAACGGCAGGT	654 bp	55 °C
	R: CGTGCCCAGTAATGGCTAAT		
