# Supplementary material for: Detection of Carbapenem Resistance of Proteus mirabilis Strains Isolated from Foxes, Raccoons and Minks in China
Source: Biology (Basel). 2022 Feb 11;11(2):292. doi: 10.3390/biology11020292 (PMC8869598; doi:10.3390/biology11020292)
Supplement: Supplementary file 1 [file biology-11-00292-s001.zip › biology-1535654-supplementary.pdf]

Table S1. Characteristics of virulence genes of 53 *P. mirabilis* isolates from fur animal farms in this study.

Strains	Farms	Sample type	*urec*	*zapA*	*pmfA*	*mrpA*	*atfC*	*atfA*	*ucaA*	*rsbA*	*rsmA*	*hmpA*
R1	raccoon	fece	+	+	+	+	+	+	+	+	+	+
R2	raccoon	fece	+	+	+	+	+	+	+	+	+	+
R3	raccoon	fece	+	+	+	+	+	+	+	-	+	+
R4	raccoon	fece	+	+	+	+	+	+	-	+	+	+
R5	raccoon	fece	+	+	+	+	+	+	-	+	+	+
R6	raccoon	fece	+	+	+	+	+	+	+	+	+	+
R7	raccoon	fece	+	+	+	+	+	+	+	+	+	+
R8	raccoon	fece	+	+	+	+	+	+	+	-	+	+
R9	raccoon	fece	+	+	+	+	+	+	-	+	+	+
R10	raccoon	fece	+	+	+	+	+	+	-	+	+	+
R11	raccoon	fece	+	+	+	+	+	+	-	+	+	+
R12	raccoon	fece	+	+	+	+	+	+	-	+	+	+
R13	raccoon	fece	+	+	+	+	+	+	-	-	+	+
R14	raccoon	fece	+	+	+	+	+	+	-	-	+	+
R15	raccoon	fece	+	+	+	+	+	+	-	-	+	+
R16	raccoon	soil	+	+	+	+	-	+	-	-	+	+
R17	raccoon	soil	+	+	+	+	+	+	-	+	+	+
R18	raccoon	soil	+	+	+	+	+	+	-	-	+	+
F1	fox	fece	+	+	+	+	+	+	+	+	+	+
F2	fox	fece	+	+	+	+	+	+	+	+	+	+
F3	fox	fece	+	+	+	+	+	+	-	-	-	+
F4	fox	fece	+	+	+	+	+	+	-	+	+	+
F5	fox	fece	+	+	+	+	+	+	-	-	+	+
F6	fox	fece	+	+	+	+	+	+	-	+	+	+
F7	fox	fece	+	+	+	+	+	+	+	+	+	+
F8	fox	fece	+	+	+	+	+	+	+	+	+	+
F9	fox	fece	+	+	+	+	+	+	-	-	+	+
F10	fox	fece	+	+	+	+	+	+	-	+	+	+
F11	fox	fece	+	+	+	+	+	+	+	+	+	+

F12	fox	fece	+	+	+	+	+	+	+	+	+	+
F13	fox	soil	+	+	+	+	+	+	+	-	+	+
F14	fox	soil	+	+	+	+	+	+	+	+	+	-
F15	fox	soil	+	+	+	+	+	+	-	+	+	+
F16	fox	soil	+	+	+	+	+	+	-	+	+	+
F17	fox	soil	+	+	+	+	+	+	+	-	+	+
F18	fox	soil	+	+	+	+	+	+	-	-	+	+
F19	fox	soil	+	+	+	+	+	+	-	-	+	+
F20	fox	soil	+	+	+	+	+	+	-	+	+	+
F21	fox	soil	+	+	+	+	+	+	+	+	+	+
F22	fox	feed	+	+	+	+	+	+	-	+	+	+
M1	mink	fece	+	+	+	+	+	+	+	+	+	+
M2	mink	fece	+	+	+	+	+	+	-	+	+	+
M3	mink	fece	+	+	+	+	+	+	+	+	+	+
M4	mink	feed	+	+	+	+	+	+	-	+	+	+
M5	mink	fece	+	+	+	+	+	+	-	+	+	+
M6	mink	fece	+	+	+	+	+	+	+	+	+	+
M7	mink	fece	+	+	+	+	+	+	-	+	+	+
M8	mink	throat swab	+	+	+	+	+	+	-	+	+	+
M9	mink	throat swab	+	+	+	+	+	+	+	+	+	+
M10	mink	throat swab	+	+	+	+	+	+	+	-	+	+
M11	mink	anal swab	+	+	+	+	+	+	+	+	+	+
M12	mink	carcass	+	+	+	+	+	+	+	+	-	+
M13	mink	carcass	+	+	+	+	+	+	+	+	-	+

“+“indicates positive ,   “–“ indicates negative.

Table S2. Characteristics of antibiotic resistance genes of 53 *P. mirabilis* isolates from fur animal farms in this study.

Strains	Farms	Sample type	*bla*$_{TEM}$	*bla*$_{CTX-M}$	*bla*$_{PSE}$	*bla*$_{SHV}$	*bla*$_{OXA-1}$	*bla*$_{OXA-23}$	*bla*$_{OXA-24}$	*bla*$_{OXA-51}$	*bla*$_{OXA-58}$	*bla*$_{OXA-48}$	*bla*$_{KPC}$	*bla*$_{NDM}$	*bla*$_{IMP}$	*bla*$_{VIM}$	*aac1*	*aac2*	*aac3*	*aadA*	*aadB*	*aphA6*	*qnrA*	*qnrB*	*qnrC*	*qnrS*	*oqxA*	*aac(6')-Ib-cr*	*sul1*	*sul2*	*sul3*	*cmlA*	*floR*
R1	rac-coon	fece	+	+	-	-	+	-	-	-	-	-	-	-	-	-	-	-	-	+	-	-	+	-	-	-	-	+	+	-	+	-	+
R2	rac-coon	fece	+	+	-	-	+	-	-	-	-	-	-	-	-	-	-	-	-	+	-	-	+	-	-	-	-	+	+	-	+	-	+
R3	rac-coon	fece	+	-	-	-	+	-	-	-	-	-	-	+	-	-	-	-	-	+	-	-	-	-	-	-	-	+	+	-	+	-	+
R4	rac-coon	fece	+	-	-	-	+	-	-	-	-	-	-	-	-	-	-	-	-	+	-	-	-	-	-	-	-	+	+	-	+	-	+
R5	rac-coon	fece	+	-	-	-	+	-	-	-	-	-	-	+	-	-	-	-	-	+	-	-	-	-	-	-	-	+	+	-	+	-	+
R6	rac-coon	fece	+	-	-	-	+	-	-	-	-	-	-	-	-	-	-	-	-	+	-	-	-	-	-	-	-	+	+	-	+	-	+
R7	rac-coon	fece	+	-	-	-	-	-	-	-	-	-	-	-	-	-	-	-	-	+	-	-	-	-	-	-	-	-	+	-	+	-	+
R8	rac-coon	fece	+	-	-	-	+	-	-	-	-	-	-	-	-	-	-	-	-	+	+	-	-	-	-	-	-	+	+	-	+	-	+
R9	rac-coon	fece	+	+	-	-	+	-	-	-	-	-	-	+	-	-	-	-	-	+	-	-	-	-	+	-	-	+	+	-	+	-	+
R10	rac-coon	fece	+	+	-	-	+	-	-	-	-	-	-	+	-	-	-	-	-	+	+	-	-	-	+	-	-	+	+	-	+	-	+
R11	rac-coon	fece	+	+	-	-	+	-	-	-	-	-	-	-	-	-	-	-	-	+	-	-	-	-	+	-	-	+	+	-	+	-	+

R12	rac-coon	fece	+	-	-	-	+	-	-	-	-	-	-	-	-	-	-	-	-	+	-	-	-	-	-	-	-	+	+	-	-	-	-
R13	rac-coon	fece	+	+	-	-	+	-	-	-	-	-	-	-	-	-	-	-	-	-	-	-	-	-	-	-	-	+	+	-	+	-	-
R14	rac-coon	fece	+	-	-	-	+	-	-	-	-	-	-	+	-	-	-	-	-	+	+	-	-	-	-	-	-	+	+	-	+	-	+
R15	rac-coon	fece	+	+	-	-	+	-	-	-	-	-	-	-	-	-	-	-	-	+	-	-	-	-	-	-	-	+	+	-	+	-	+
R16	rac-coon	soil	+	+	-	-	+	-	-	-	-	-	-	{Ahn, 2017 #67}	-	-	-	-	-	+	+	-	-	-	-	-	-	+	+	-	+	-	+
R17	rac-coon	soil	+	+	-	-	+	-	-	-	-	-	-	-	-	-	-	-	-	+	-	-	-	-	+	-	-	+	+	-	+	-	+
R18	rac-coon	soil	+	+	-	-	+	-	-	-	-	-	-	-	-	-	-	-	-	+	-	-	-	-	+	-	-	+	+	-	+	-	+
F1	fox	fece	+	-	-	-	+	-	-	-	-	-	-	-	-	-	-	-	-	+	-	-	-	-	-	-	-	-	+	-	-	-	+
F2	fox	fece	+	-	-	-	-	-	-	-	-	-	-	-	-	-	-	-	-	+	-	-	-	-	-	-	-	-	+	-	-	-	+
F3	fox	fece	+	-	-	-	-	-	-	-	-	-	-	-	-	-	-	-	-	-	-	-	-	-	-	-	-	-	+	-	-	-	+
F4	fox	fece	+	-	-	-	-	-	-	-	-	-	-	-	-	-	-	-	-	-	-	-	-	-	-	-	-	+	+	-	-	-	+
F5	fox	fece	+	+	-	-	-	-	-	-	-	-	-	-	-	-	-	-	-	+	-	-	-	-	-	-	-	-	+	-	+	-	+
F6	fox	fece	+	-	-	-	-	-	-	-	-	-	-	-	-	-	-	-	-	-	-	-	-	-	-	-	-	+	+	-	-	-	+
F7	fox	fece	+	+	-	-	+	-	-	-	-	-	-	-	-	-	-	-	-	-	-	-	-	-	+	-	-	-	+	-	+	-	+
F8	fox	fece	+	+	-	-	-	-	-	-	-	-	-	-	-	-	-	-	-	-	-	-	-	-	-	-	-	-	+	-	-	-	+
F9	fox	fece	+	-	-	-	-	-	-	-	-	-	-	-	-	-	-	-	-	+	-	-	-	-	-	-	-	-	+	-	-	-	+
F10	fox	fece	+	-	-	-	-	-	-	-	-	-	-	-	-	-	-	-	-	-	-	-	-	-	-	-	-	-	+	-	-	-	+
F11	fox	fece	+	+	-	-	+	-	-	-	-	-	-	-	-	-	-	-	-	+	-	+	-	-	-	-	-	+	+	-	+	-	+
F12	fox	fece	+	-	-	-	-	-	-	-	-	-	-	-	-	-	-	-	-	+	-	+	-	-	-	-	-	-	+	-	+	-	+
F13	fox	soil	+	-	-	-	-	-	-	-	-	-	-	-	-	-	-	-	-	-	-	+	-	-	-	-	-	-	+	-	-	-	+
F14	fox	soil	+	-	-	-	-	-	-	-	-	-	-	-	-	-	-	-	-	+	-	-	-	-	-	-	-	-	+	-	-	-	+
F15	fox	soil	+	+	-	-	+	-	-	-	-	-	-	-	-	-	-	-	-	+	-	-	-	-	-	-	-	+	+	-	+	-	+

F16	fox	soil	+	-	-	-	-	-	-	-	-	-	-	+	-	-	-	-	-	+	+	-	-	-	-	-	-	+	+	-	+	-	+
F17	fox	soil	+	-	-	-	-	-	-	-	-	-	-	-	-	-	-	-	-	+	-	-	-	-	+	-	-	-	+	-	-	-	+
F18	fox	soil	+	-	-	-	-	-	-	-	-	-	-	-	-	-	-	-	-	+	-	-	-	-	-	-	-	-	+	-	-	-	+
F19	fox	soil	+	-	-	-	-	-	-	-	-	-	-	-	-	-	-	-	-	+	-	-	-	-	-	-	-	-	+	-	+	-	+
F20	fox	soil	+	-	-	-	-	-	-	-	-	-	-	-	-	-	-	-	-	+	-	-	-	-	+	-	-	-	+	-	+	-	+
F21	fox	soil	+	-	-	-	-	-	+	-	-	-	-	-	-	-	-	-	-	+	-	-	+	-	-	-	-	+	+	-	+	-	+
F22	fox	feed	+	+	-	-	+	-	+	-	-	-	-	-	-	-	-	-	-	+	-	-	-	-	-	-	-	+	+	-	+	-	+
M1	mink	fece	-	-	+	-	+	-	+	-	-	-	-	-	-	-	-	-	-	+	-	-	-	+	-	-	-	+	+	-	+	+	+
M2	mink	fece	-	-	+	-	+	-	+	-	-	-	-	-	-	-	-	+	-	+	-	-	-	+	-	-	-	+	-	+	-	-	-
M3	mink	fece	-	-	+	-	+	-	-	-	-	-	-	-	-	-	-	-	-	+	-	-	-	+	-	-	-	+	+	+	-	-	-
M4	mink	fece	-	-	+	-	+	-	+	-	-	-	-	-	-	-	-	+	-	+	-	-	-	+	-	-	-	+	+	-	-	-	+
M5	mink	fece	+	-	+	-	+	-	-	-	-	-	-	-	-	-	-	-	-	+	-	-	-	+	-	-	-	+	+	+	-	+	+
M6	mink	fece	-	-	+	-	+	-	-	-	-	-	-	-	-	-	-	+	-	+	+	-	-	+	-	-	-	+	+	+	-	-	-
M7	mink	feed	-	-	+	-	+	-	+	-	-	-	-	-	-	-	-	+	-	-	-	-	-	+	-	-	-	+	-	-	-	-	+
M8	mink	throat swab	-	-	+	-	+	-	-	-	-	-	-	-	-	-	-	+	-	+	+	-	-	+	-	-	-	+	-	+	-	-	-
M9	mink	throat swab	-	-	+	-	-	-	-	-	-	-	-	-	-	-	-	-	-	+	-	-	-	+	-	-	-	+	+	-	-	-	+
M10	mink	throat swab	-	-	+	-	-	-	+	-	-	-	-	-	-	-	-	-	-	+	-	+	-	+	-	-	-	+	+	+	-	-	+
M11	mink	anal swab	+	-	+	-	+	-	-	-	-	-	-	-	-	-	-	-	-	+	-	+	-	+	-	-	-	+	+	-	-	-	+
M12	mink	carcass	+	-	+	-	+	-	+	-	-	-	-	-	-	-	-	-	-	+	-	+	-	-	-	-	-	+	+	-	-	+	-
M13	mink	carcass	+	-	+	-	-	-	-	-	-	-	-	-	-	-	-	-	-	+	-	-	-	+	-	-	-	+	+	-	-	-	-

“+“indicates positive , “–“ indicates negative.
